# Wear and Airborne Noise Interdependency at Asperitical Level: Analytical Modelling and Experimental Validation

**DOI:** 10.3390/ma14237308

**Published:** 2021-11-29

**Authors:** Kevin Lontin, Muhammad A. Khan

**Affiliations:** 1 School of Aerospace, Transport and Manufacturing, Cranfield University, Bedford MK43 0AL, UK; 2 Centre for Life-Cycle Engineering and Management, Cranfield University, Bedford MK43 0AL, UK; Muhammad.A.Khan@cranfield.ac.uk

**Keywords:** friction, wear, airborne noise, asperities, analytical model

## Abstract

Generation of wear and airborne sound is inevitable during friction processes. Previously, the relationship between the wear and the sound has only been determined experimentally. Analytical models do exist, but they remain rare and do not fully account for the wear and the airborne sound generation especially at the asperitical level. This model attempts to fill the gap by providing a quantifiable relationship at an asperitical level between the wear generated and the sound emitted in a simple pin-on-disc setup. The model was validated for three materials (iron, mild steel, and aluminium T351) under two loads (10 N and 20 N) at 300 RPM. The theoretical model agrees with the experimental results with a varying error of 10 to 15% error in iron and aluminium. However, a larger error is observed in the case of mild steel. The model could be refined to improve the accuracy as it assumes point impacts on the asperities where a distributed impact would be more suitable. Furthermore, the pin is assumed a single asperity to simplify the model at the expense of accuracy. Overall, the experimental results are in good correlation with the theoretical results and this model provides the first step in quantifying wear using only the recorded sound pressure.

## 1. Introduction

Wear processes present a significant challenge in industry. This is because wear reduces the useful life of machine components. This leads to expensive component replacement [1]. The friction of worn-out surfaces of these components influences the wear processes. The mechanism of these processes depends on the mechanical properties and the physical geometries of the surfaces in contact and the load applied [2]. When degradation occurs due to the surface wear, the emitted sound will change due to the release of energy from the localized sources within the material [3]. This is because sound is emitted in any friction process. During a friction process, energy is transferred from one surface to another while energy dissipation (in the form of heat) occurs. Instabilities are created when the energy supplied is larger than the energy dissipated. Those instabilities caused by the friction process are what lead to the generation of sound. They are caused by vibrations induced by the frictional system which leads to harmonic oscillations of the friction pair at their fundamental frequencies. This means that analysing the spectrum of the sound emission can lead to an understanding of the wear and friction processes that occurred within the material. There have been a lot of experimental observations of the change of the sound spectrum emitted by materials undergoing a friction process, and those changes have been linked to the wear. For example, Finkin examined how the surface roughness changes due to the wear phenomenon [4] and Yokoi and Nakai correlated the influence of the surface roughness on the generation of sound on a pin-on-disc experiment [5]. It was found that as the surface roughness increased, the sound pressure level also increased. The transfer of material between two contacting surfaces under wear along with the deformation of the materials was investigated by Jacobson et al. [6]. Numerical models were also developed to examine the interdependencies between the sound and the friction processes. Abdelounis, et al. determined that the sound was generated by the impacts between the antagonist asperities across the surface which then converted the kinetic energy of the impact to vibrational energy which was responsible for the radiation of sound [7]. In their studies, however, the asperities were assumed to be vibrating elastically. The vibrational energy caused by the impact would be translated to sound pressure. This did not account for the wear. Other numerical studies were performed to analyse brake squeal generation, which is something important in the automotive industry [8]. Furthermore, a lot of research also involved the study of acoustic emissions as opposed to airborne noise. It is possible to link wear and acoustic emissions in the same way as to wear and noise. Boness and McBride performed a study of the change in acoustic emissions during a wear process [9]. They also studied how lubrication would affect acoustic emission [10]. Acoustic emissions can also be used to predict airborne noise. Benabdhallah and Aguilar investigated the effect of acoustic emission on airborne noise [11]. However, analytical models encompassing acoustic emissions and their relationship with both wear and airborne noise are missing from the literature and still need to be developed. As for analytical friction models, a theoretical asperity-based model for friction processes was also developed by De Moerlooze et al. however, their model does not account for the wear either [12]. More advanced friction models such as the one by Eriten et al. [13] or Emami et al. [14] are asperity-based but do not account for the wear. Numerical models for elastoplastic contact interfaces were also developed but do not take sound generation into account [15].

There have also been numerous wear models that have been developed, however, their linkage with advanced friction models is weak and most of them are purely empirical. For example, Savio et al. created an empirical model for sliding wear [16], whereas Quinn created an empirical model for oxidational wear [17]. More recently, numerical, and theoretical models were developed. However, none of them takes into account airborne noise or acoustic emissions. For example, Shen et al. developed a numerical model in ABAQUS for sliding wear monitoring [18]. Hassan and Mohammed investigated sliding wear using a neural network for wear prediction [19]. An analytical model for wear processes was developed by Filliot et al. [20]. This model introduced the third-body concept for wear debris. However, it did not account for the contribution of the third body to the sound generation.

This means that there is no general analytical model that unifies the sound and the wear during friction processes. This paper attempts to fill that gap by providing an asperity-based model that takes the wear into account and predicts the sound generation. This is necessary because as wear occurs and asperities are broken off, the energy caused by the breakage of the asperities is converted to sound energy. This model is the first step to provide a non-destructive method of predicting the wear occurring during a friction process by analysing the sound pressure under dry conditions.

## 2. Methodology

### 2.1. Analytical Modelling

#### 2.1.1. Theory and Assumptions

**Figure 1:**
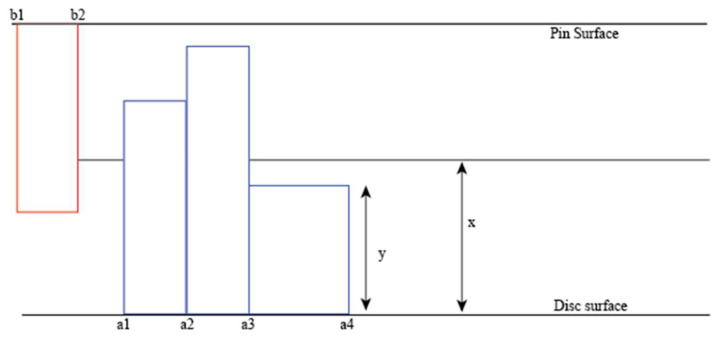
Conditions of contact.

The analytical model is created to quantify the interdependence of sliding wear and sound emitted in a pin-on-disk setup as shown in Figure 1. *a*1, *a*2, *a*3, *a*4 lie on a plane on the bottom surface (surface of the disc), *b*1, *b*2, lie on a plane on the top surface (tip of the pin), *y* is the height of an asperity and *x* is the distance from either surface to the centreline axis (located at a mid-distance between the two surfaces). As the asperities on the disc are struck by the asperities on the pin, the sound can be emitted due to two mechanisms:

*Mechanism-1*: If the asperities undergo elastic deformation, then the sound is emitted due to the elastic vibration of the asperities [21]. However, some further assumptions were made for elastic vibrations. For this kind of vibrations, there can be several dynamic instabilities contributing to the generation of sound. For instance, mode-coupling instability can lead to acoustic propagation. Furthermore, a beam will vibrate in all modes. However, it is found that the first mode of vibration will have the largest impact on the displacement and the sound generation as it is the mode that contains the most energy. The other modes end up having a negligible contribution to the sound generation as they contain much less energy. As such, only the first mode was considered for the vibration of the beams.

*Mechanism-2*: If the asperities break under the force of the impact [22], then the mechanism for the generation of sound will be different. This is due to the differences in asperity lengths. In mechanism 1, the asperity length will be higher, and this induces a lower flexural stiffness. Similarly, for mechanism 2, the flexural stiffness is higher. As such, the vibration frequencies will be different and so will the sound pressure.

We assume that if the asperities undergo plastic deformation, then no sound is emitted. This is because all the energy is used in the deformation. We also assume that asperities that undergo plastic deformation do not go back to the elastic zone anymore. It is possible for asperities that undergo plastic deformation to still display some elastic behaviour in the next cycle. However, that elastic behaviour will be smaller than in the previous cycles and so their contributions to the noise will be more limited in subsequent cycles and so it was decided to neglect them. Furthermore, elastoplastic behaviour was neglected to reduce the complexity of the model. This is because elastoplasticity would provide a negligible contribution to either the noise or the wear compared to a fully elastic asperity (which contributes to the noise due to the vibration) and the plastic deformation (which leads to the wear). Asperities that are in elastoplastic deformation do not remain in that state for long due to the speed of the cycles. They would quickly enter the plastic stage and eventually break. As there are no compression waves generated during plastic deformation, the asperities do not vibrate. To account for those mechanisms, the model is divided into three components. First, the model generates a distribution of heights for the asperities on the surface of the disk. To prevent the model from being computationally prohibitive, only one asperity on the pin is modelled. This is because it is assumed that the pin would have negligible wear. For that to hold, the pin is replaced between each experiment. The distribution of heights on the disc asperities is assumed to be exponential as this gives a good approximation to the actual distribution of asperities [23]. As the pin strikes each asperity during the rotation of the disc, the stresses are calculated for each impact. This determines whether the asperities will be elastically vibrating, plastically deformed, or broken. This is used to determine which mechanism of sound is used to calculate the sound generated by the struck asperity. Furthermore, as wear occurs on the disc, the asperitical map changes and is replaced after each rotation by a new asperitical map. To this effect, a wear function is also developed to calculate the changes in the asperitical map. It should be noted that other friction models use a counter-profile that the pin asperity would slide over. However, modelling asperities as a continuous counter-profile would involve high computational costs. Therefore, the interactions were made in the form of impacts as it provides a reasonable starting postulate for the model while keeping the computational cost acceptable.

When the asperities are in contact, three conditions can occur. The two asperities can be either elastically deformed, plastically deformed, or just in contact:

*Condition 1*: If the average length of two contacting asperities equals the distance to the centreline, then the two asperities will just be in contact:

$$\frac{y_{a_{n}}+\;y_{b_{n}}}{2}=x$$

*Condition 2*: If it is greater than the centreline, then the asperities will be deformed. If the deformation caused by the stress is less than the elastic limit, then elastic deformation will occur:

$$\frac{y_{a_{n}}+\;y_{b_{n}}}{2}\;>x$$

*Condition 3:* If the stress caused by the deformation is greater than the elastic limit, then plastic deformation will occur.

$$\frac{y_{a_{n}}+y_{b_{n}}}{2}\;\gg x$$

Whether the deformation is plastic or elastic depends on the material properties such as the elastic modulus and the yield strength. It is also assumed that all the asperities are independent of one another. This means that what happens to the previous asperity does not influence the next asperity. This assumption was made even though when an asperity on the disc is struck, it can set off several collisions between neighbouring asperities. However, the complexity of the model drastically increases when that happens. Neighbouring asperities may start vibrating too, which can set off even further collisions. Vibrations could be constructive leading to an increase in sound, or they may vibrate in the opposite direction thus cancelling each other out, leading to a decrease in sound. The high number of uncertainties in such situations led to the simplification that the distance between each asperity on the disc was sufficient that interactions would not occur. Furthermore, it is assumed that the pin asperity does not wear out. This is because, during the experimental validation, the pin is replaced after each experiment and wear is not observed.

#### 2.1.2. Deriving the Wear Function

**Figure 2:**
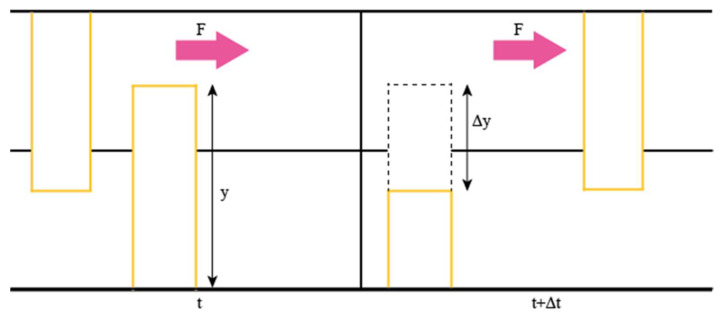
Height changes of an asperity under wear.

As the pin undergoes no wear, the wear function does not have to be applied to the pin. The impacts between the top asperity and the bottom asperities are represented as point impacts. Wear occurs when the impact between the asperities lead to deformation such that the total stress on the asperity exceeds the ultimate tensile strength which leads to the asperities breaking off. As such, wear can be taken as a function of deformation only *W*(*y* − *x*). It should be noted that the deformation of interest is due to the tangential force only. This is because the normal force will not contribute to the sound generation. This means that an asperity at a height of *y* at a time *t*, will be at a height of *dy* = *y* − Δ*y* at time *t* + Δ*t* under wear as shown in Figure 2:

This can be summarised in the following two equations:

(1) $$\frac{\Delta y}{\Delta t}=W\left(y\;-x\right)\;\mathrm{so}\;\Delta y=W\;\left(y-x\right)\Delta t$$

(2) dy=y−Δy so dy=y−W(y−x)Δt

Differentiating with respect to y to get the rate of change of the wear gives:

(3) $$\frac{d^{\prime }y}{dy}=1-W^{\prime }\left(y-x\right)\Delta t$$

(4) $$d^{\prime }y=dy\left(1-W^{\prime }\left(y-x\right)\Delta t\right)\;$$

The height distributions of the asperities can be given as *Φ* (*y*, *t*) at any time *t*. At any time, *t* + Δ*t*, the asperities will have a height of dy. This is given by the new distribution *Φ* (*y* − Δ*y*, *t* + Δ*t*) where Δt is the time step. In the case of the pin-on-disc setup, the time step is the time between each consecutive contact on the same asperity (which is the time for one revolution). Multiplying the original roughness distribution by the wear function gives the new distribution function as shown in Equation (5):

(5) W(y−x)∗Φ(y,t)=Φ(y−Δy, t+Δt)

By corollary, this means that dividing the new distribution function by the wear function returns the original distribution function as shown in Equation (6):

(6) $$\Phi \left(y,t\right)=\Phi \left(y-\Delta y,\;t+\Delta t\right)*\;\frac{1}{W\left(y-x\right)}$$

The changes in the height of each asperity are also linked to the wear function so that Equation (7) holds:

(7) $$d^{\prime }y=dy*W\left(y-x\right)$$

This equation can be rearranged in the form of Equation (8):

(8) $$\frac{1}{W\left(y-x\right)}=\;\frac{dy}{d\prime y}$$

The relationship between the original distribution function and the subsequent distribution function can therefore be expressed in the form of Equation (9) by combining Equations (6) and (8):

(9) $$\Phi \left(y,t\right)=\Phi \left(y-\Delta y,\;t+\Delta t\right)\frac{dy}{d^{\prime }y}$$

Rearranging gives:

(10) $$d^{\prime }y\Phi \left(y,t\right)=\Phi \left(y-\Delta y,\;t+\Delta t\right)dy$$

With the relationship established, a partial differential equation can be formed to relate the asperity height to the wear of the material. This is done by taking the Taylor expansion of the function.

(11) $$dy\left(1-W^{\prime }\left(y-x\right)\Delta t\right)\Phi \left(y,t\right)=dy\Phi \left(y,t\right)-dy\Delta y\frac{\partial }{\partial y}\Phi \left(y,t\right)+dy\Delta t\frac{\partial }{\partial t}\Phi \left(y,t\right)$$

This is a first-order Taylor expansion. This equation can be rearranged and then simplified by expanding the left-hand side, dividing by d*y*Δ*t*, and subtracting *Φ*(*y*,*t*). Applying the reverse chain rule gives:

(12) $$\frac{\partial }{\partial t}\Phi \left(y,t\right)=-(W\left(y-x\right)\Phi^{\prime }\left(y,t\right)+W^{\prime }\left(y-x\right)\Phi \left(y,t\right)\;$$

(13) $$\frac{\partial }{\partial t}\Phi \left(y,t\right)=-\frac{\partial }{\partial y}\left(W\left(y-x\right)\Phi \left(y,t\right)\right)$$

To solve the partial differentiation equation, a substitution u is used, *where u* = log(*Φ*(*y*,*t*)):

(14) $$\frac{\partial u}{\partial t}=\;\frac{\Phi \prime \left(y,t\right)}{\Phi \left(y,t\right)}\;\;\mathrm{so}\;\frac{\partial u}{\partial t}\Phi \left(y,t\right)=-W\left(z\right)\frac{\partial u}{\partial y}\Phi \left(y,t\right)-W^{\prime }\left(z\right)\Phi \left(y,t\right)$$

$$\frac{\partial u}{\partial t}+W\left(z\right)\frac{\partial u}{\partial y}=-W\prime \left(z\right)\;\mathrm{when}\;z=y-x$$

(15) $$dt=\frac{dy}{W\left(z\right)}=-\frac{du}{W^{\prime }\left(z\right)}$$

Changing variable gives dz=dy so $dt=\;\frac{dz}{W\left(z\right)}$ and since $du=-W^{\prime }\left(z\right)dt$, then $du=-\frac{W\prime \left(z\right)}{W\left(z\right)}dz$.

The general solution can be expressed as:

$$f\left(u,y,t\right)=\;c_{1}\;\mathrm{and}\;g\left(u,y,t\right)=\;c_{2}\;f=\varphi \left(z\right)-t=\;c_{1},\;\mathrm{where}\;\varphi \left(z\right)=\;\int_{0}^{z}\frac{1}{W\left(z\right)}dz$$

(16) $$g=u+log\left[W\left(z\right)\right]=c_{2}$$

At t=0, let G(y)=log[Φ(y,0)]:

(17) u −G(y)=0

If we use the inverse function to express the deformation as a function, then:

Let θ(φ(z))= z

(18) y=x+θ(f)

u=g−log[W{θ(f)}] so g−log[W{θ(f)}]−G(x+θ(f))=0

The final solution is therefore:

(19) u=−log[W(z)]+log[W{θ(φ(z)−t)}]+G[x+θ(φ(z)−t)]

A relationship between asperitical height and wear is found:

(20) $$\Phi \left(y,t\right)=\;\frac{W\left[\theta \left\{\varphi \left(z\right)-t\right\}\right]}{W\left(z\right)}exp\left(G\left[x+\theta \left(\varphi \left(z\right)-t\right)\right]\right)$$

Each quantity is defined as follows:

*Φ* is the asperitical height distribution function

*W* is the wear function:

$$\varphi =\;\int^{}\frac{1}{W\left(z\right)}\;dz$$

*θ* is the inverse function such that *θ*(*φ*(*z*)) = *z*

*G* = log(*Φ*(*y*,0))

#### 2.1.3. Calculating the Stresses on the Asperities Caused by the Impacts of the Pin

To apply this relationship to the case of the pin sliding on the disc, an appropriate wear function must be found. A fundamental assumption that is made to simplify the wear function equation is that the asperities are modelled as beams and thus follow the beam equations. Furthermore, the scale of the asperitical contacts is large enough so that the equations valid for macro-structures are also valid for the asperities. This is because the apparent area of contact is much larger than the scale of the asperities. Micro-tribology would only be required if the scale of the apparent area of contact was of the same order of magnitude as the scale of the asperities themselves [24]. The scale of the apparent and real area of contact is important for modelling. For example, dry conditions are no longer dry if the scale becomes small enough. This is because ambient humidity would cause water droplets to form a thin layer between the two surfaces. This effect is ignored in our model. First, the impact force on one asperity is calculated using the following equation:

(21) Ft=mv

where *F* is the impact force, *t* is the contact time, *m* is the mass of the asperity and *v* is the velocity of the disc. The contact time can be determined by the total number of asperities. If the pin asperity contacts all opposing asperities for the same length of time, the contact time can be determined using the following equation:

(22) $$t=\;\frac{T}{N}$$

where *T* is the total time taken for one rotation and *N* is the total number of asperities. The mass of an asperity can be calculated using the following equation:

(23) m=pV

where *p* is the density of the material and *V* is calculated as follows:

(24) V=cH

where *c* is the cross-sectional area of the asperity and *H* is the height.

**Figure 3:**
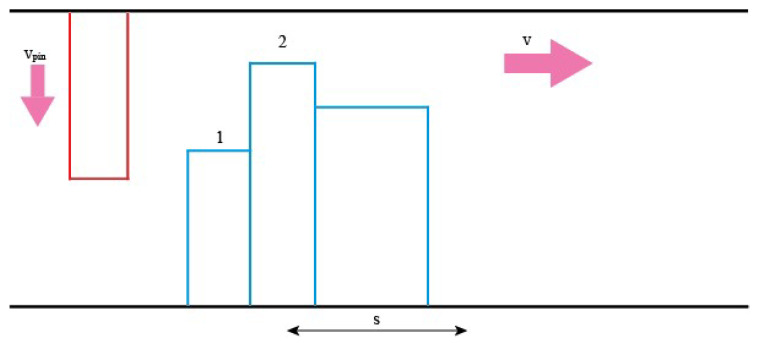
V is the relative velocity of the pin. s is the width of an asperity. *TBC* is the time taken for the pin to go from position 1 to position 2.

The total number of asperities present on the disc depends on the width of each asperity. Assuming an exponential distribution of width, similarly to the height distribution gives us the necessary information. The sum of the widths of the asperities (until the total distance travelled by the pin in one rotation is reached) gives the number of asperities. The force of impact on each asperity can then be calculated. From this, the maximum bending moment can also be calculated. The point of impact on the asperity must also be calculated. It is done as follows:

The asperity on the pin is under positive load, it moves down at a constant acceleration as it carves the wear track on the disc as shown in Figure 3. The disk asperities also travel at a constant horizontal velocity. The linear velocity of the disk can be calculated. Knowing the velocity of the disk and the width distribution of the asperities, the time between contacts (hence the time the pin must travel down before reaching the next asperity) can be calculated as follows:

(25) $$TBC=\;\frac{s}{v}$$

where *TBC* is the time between contacts, and *s* is the width of the asperity. As for the pin, its downwards velocity as it reaches the next asperity can be calculated as follows:

(26) $$v_{pin}=u+g*TBC$$

where *v_pin_* is the initial velocity of the pin at the next point of contact, *u* is the initial velocity at the previous point of contact, *g* is the acceleration and *TBC* is the time between contacts. With the velocity calculated, the downwards displacement of the pin can be calculated. This determines if the pin has enough time to meet the next asperity and if so, at what point:

(27) $$s_{pin}=ut+\frac{1}{2}g*TBC^{2}$$

If the height difference between two successive asperities is less than the distance travelled by the pin, then contact is made, and a (in Equation (26)) is equal to *H* − *s_pin_* and thus, the maximum stress is given by the following expression:

(28) $$\theta =\;\frac{M}{S}$$

where *S* is the section modulus which will depend on the cross-section of the asperity.

The maximum stress can then be compared to the maximum tensile strength of the material. For each asperity, if the stress exceeds the strength of the material, the asperity will break, and wear will occur. If it does not but exceeds the yield strength, then the material will be plastically deformed. If neither of those conditions is achieved, then the asperity will be elastically vibrating.

#### 2.1.4. Sound Produced Due to Elastically Vibrating Asperities

To calculate the sound produced due to an elastically vibrating asperity, its displacement is first calculated using the following equation:

(29) D(x,t)=X(x)∗ [Acos(ωt)+B sin(ωt)]

where *ω* is the angular frequency. The displacement is both time-dependant and position-dependant. According to Volterra’s dynamics of vibration [25], *X*(*x*) is given by:

$$X\left(x\right)=\;\frac{1}{2}\left\{\left[\cos\left(\lambda x\right)-\;\cosh\left(\lambda x\right)\right]+\;\left[\frac{-\cos\left(\lambda L\right)-\;\cosh\left(\lambda L\right)}{\sin\left(\lambda L\right)-\;\sinh\left(\lambda L\right)}\right]\left[\sin\left(\lambda x\right)-\;\sinh\left(\lambda x\right)\right]\right\}$$

The constants *A* and *B* must be found. *A* depends on the initial position at time *t* = 0. *B* depends on the initial velocity. *B* = 0 because the asperity is not vibrating at time *t* = 0. *At X* = *L*, *X*(*L*) = 1. *A* is given in the following equation:

$$A=\;\frac{2}{L}\overset{L}{\underset{0}{\int }}D\left(x,\;t=0\right)X\left(x\right)\;dx$$

By solving this equation, *A* is found to be given by Equation (30):

(30) $$A=\;\left[\frac{-4WL}{EI\rho A\lambda^{4}\left(\lambda L\right)e^{\lambda L}+e^{2\lambda L}-1)}\right]*\left[3sin\left(\lambda L\right)\left(e^{2\lambda L}+1\right)-2{\left(\lambda L\right)}^{3}e^{\lambda L}+cos\left(\lambda L\right)\left(3-{\left(\lambda L\right)}^{3}\left(e^{2\lambda L}+1\right)-3e^{2\lambda L}\right)\right]$$

where *λ* is a coefficient given by the following equation:

(31) λ= (ρAEIω2)14

The displacement, in this case, corresponds to the deflection of the beam. *E* is the elastic modulus, and *I* is the moment of inertia.

If the system is underdamped (as it would be), then the damping ratio can be calculated using the following equation:

First, the critical damping is determined by:

(32) $$c_{c}=\;2m\omega$$

The damping coefficient can be calculated as follows:

(33) $$C=4bL\sqrt{\frac{2m}{\pi k_{b}T}P}$$

where *b* is the beam width, *k_b_* is the Boltzmann constant, *T* is the ambient temperature and *P* is the ambient pressure.

The damping ratio is then calculated as follows:

(34) $$\xi =\;\frac{C}{c_{c}}$$

The damped displacement can be calculated as follows:

(35) $$X=De^{-\xi \omega t}cos\left(\omega t\right)$$

where *t* is the time of the next impact (which is the time of one rotation)

**Figure 4:**
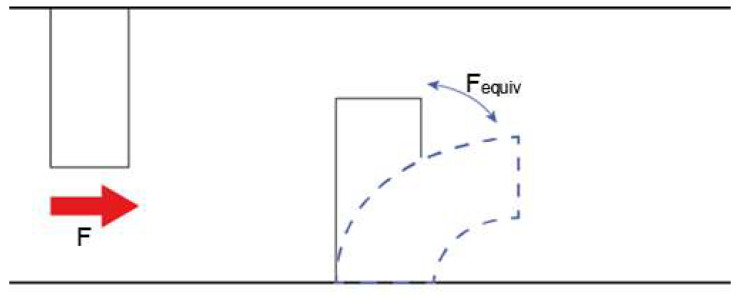
F is the impact force caused by the top asperity. F_equiv_ is the force generated by the vibration of the bottom asperity due to the previous impact.

On subsequent cycles, asperities that were already struck in the preceding cycle would still be vibrating due to the impact. An equivalent impact force can be calculated using deflection equations [26]. The equivalent impact force is the force that the vibration of the asperity is inducing, as shown in Figure 4:

The equivalent impact force can then be calculated by:

(36) $$F_{equiv}=\;\frac{6XEI}{a^{2}\left(3L-a\right)}$$

The moment and stress can thus be calculated as before by adding the equivalent impact force and the true impact force together as shown in Equation (37). This is because the total force would be the sum of the force induced by the vibrating asperity as it was struck in the previous cycle and the force of impact caused by the next impact of the pin on the already vibrating asperity in the next cycle:

(37) $$F_{Total}=F+\;F_{equiv}$$

The vibrational velocity of an elastically vibrating asperity can be calculated as follows:

(38) $$V=\;-\xi \omega tDe^{-\xi \omega t}cos\left(\omega t\right)-De^{-\xi \omega t}sin\left(\omega t\right)$$

The acoustic power can then be calculated using the following equation:

(39) $$P=\;\rho_{0}cS\sigma V^{2}$$

where *ρ*_0_ is the air density, *c* is the speed of sound in air, *S* is the cross-sectional surface area, *σ* is the radiation efficiency, and *V* is the vibrational velocity [27]. The sound power level is given by the following equation:

(40) $$L_{w}=10\;{log}_{10}\left(\frac{P}{P_{r}}\right)$$

where *P_r_* is the reference power (=10^−12^ W). The sound pressure level is given by the following equation:

(41) $$L_{p1}=\;L_{w}+10\;{log}_{10}\left(\frac{Q}{4\pi r^{2}}\right)$$

where *Q* is the directivity factor. It could be either 1 (full sphere propagation), 2 (half-sphere propagation), 4 (quarter sphere propagation) or 8 (eighth sphere propagation). The sound pressure can then be calculated using the following equation:

(42) $$P=\;P_{0}*10^{\frac{1}{20}L_{p1}}$$

where *P*_0_ is the reference pressure (20 × 10^−6^ Pa). 

#### 2.1.5. Sound Produced Due to Breaking Asperities

**Figure 5:**
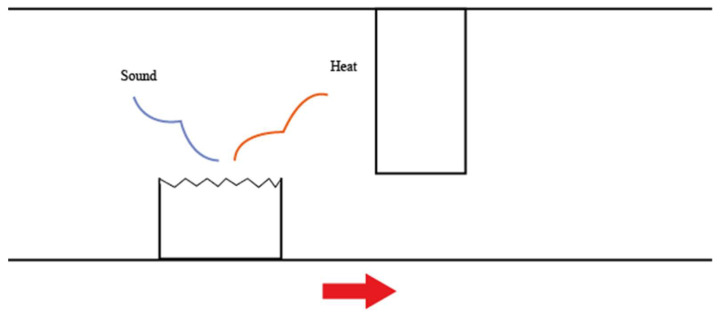
Sound and heat are released as the asperity breaks.

If the asperities are broken, the kinetic energy caused by the impact between the two asperities is converted to sound and heat energy (as shown in Figure 5):

The energy can be calculated using the following equation [28]:

(43) $$\gamma =\;H\pi r^{3}\beta$$

where *H* = hardness, and *β* = 0.5 (if half of the energy is converted into sound and the rest is converted into heat). The sound power is related to the energy as given by the following equation:

(44) P=Acγ

where *A* is the cross-sectional area of the asperity and *c* is the speed of sound in air. The sound power level, the sound pressure level and the sound pressure can be calculated using the previous equations. The total pressure level (combining the elastic vibration of one asperity and the breaking of one asperity) can be calculated using the following equation:

(45) $$L_{total}=10{log}_{10}\left(\frac{L_{p1}{}^{2}+L_{p2}{}^{2}}{L_{0}{}^{2}}\right)$$

where *L*_0_ is the reference pressure level (=2 × 10^−5^ Pa) and *L*_p2_ is the pressure level caused by breaking off one asperity.

**Figure 6:**
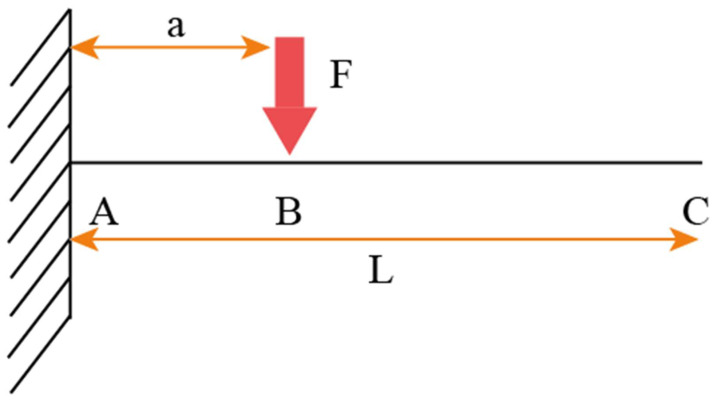
Forces acting on a cantilever beam.

In the case of breaking asperities, once the asperity has broken off (assuming it breaks off at its base), a new asperity is generated at its place, creating a new asperitical map, the new asperitical map must be determined using Equation (20). However, the wear function must be calculated. Under the assumption that the asperity behaves as a cantilever beam allows us to calculate the stress and the deflection of the asperity when acted on by a point force. This is shown in Figure 6:

Where A is the point of the asperity located on the substrate, C is the endpoint of the asperity and B is the point at which the impact occurs. The total critical deflection at C is the sum of the deflections from A to B and the deflection from B to C. The critical deflection from A to B is given as follows:

(46) $$z=\;\frac{Fa^{3}}{3EI}$$

This can be rewritten as:

(47) $$z=\;\frac{\theta Sa^{2}}{3EI}$$

From there, we can establish a function *a*(*z*):

(48) a(z)= (3EIθS)12z12

The critical deflection from B to C is given as follows:

(49) $$z=\;\frac{3Fa^{3}b}{6EIa}$$

Similarly, a function *b*(*z*) can be established:

(50) $$b\left(z\right)=\frac{2EI}{\theta Sa\left(z\right)}z$$

Substituting for *a*(*z*)*,* we get:

(51) b(z)= 2EIθS(3EIθS)12z12

Since we are assuming that the asperity breaks off at its base, then *W*(*z*) = *a*(*z*) + *b*(*z*), so:

(52) W(z)=Az12+Bz12

where:

(53) A= (3EIθS)12

(54) B=2EIθS(3EIθS)12

This can be rewritten as:

(55) W(z)= Cz12

where *C* = *A* + *B*.

Using this wear function and substituting it in the partial differential equation allows us to generate a new asperitical map when the previous one is broken off. This is repeated after each cycle to calculate the total sound and wear. It should be noted that the effect of wear debris has been neglected during the generation of the new asperitical map. The effect of wear debris would be hard to incorporate analytically since their effect on friction noise can be either positive or negative and reports on this matter are conflicting. However, due to the speed at which the disc spun, the wear debris was mostly propelled away from the wear track and the wear tracks looked mostly free of debris. As such, it was felt reasonable that the wear debris would not be incorporated into the new asperitical map.

## 3. Experimental Setup

**Figure 7:**
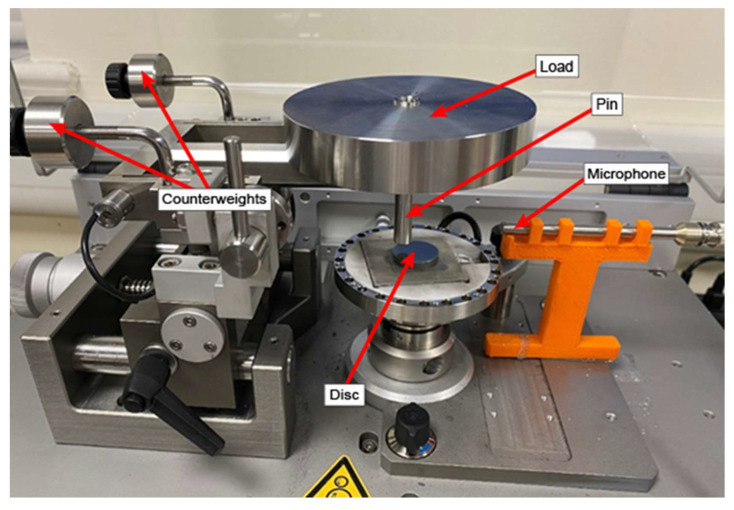
Experimental setup. The microphone is placed next to the rotating disc. It is connected to an acquisition card, itself connected to a computer.

To validate the above analytical approach, pin-on-disc experiments are conducted on various materials at constant sliding speeds under varying load conditions. No lubrication is used in these experiments. The experimental setup is shown in the following Figure 7:

A stationary 3 mm radius pin made from stainless steel (440C) is set up on a rotating disc. Only one pin is used in the experiment. A GRAS pressure microphone with a maximum operating frequency of 20 kHz is used to record the sound emitted by the tribometer due to the friction process. The microphone is placed at around 10 cm away from the pin and the disc. The microphone is connected to a NI 9350 DAQ card, which is connected to a computer. The disc samples consist of three materials: Iron, mild steel and aluminium T351. The test is performed at a speed of 300 RPM under two different loads for 10 min: 10 N and 20 N. Each test is repeated 3 times and the average value for the wear and the sound is taken. The acquisition rate on the DAQ card is set at 25.6 kHz. The sensitivity of the microphone was set at 47.46 mV/Pa. The wear is calculated by using the penetration depth sensor on the tribometer and multiplying it with the distance travelled by the pin in that timeframe. The sampling frequency on the penetration depth sensor is 100 Hz. The accuracy of the measurements is ±0.003 Pa for the sound pressure and the accuracy of the measurements for the wear are ±1 × 10^−5^ mm^3^. The data for the sound pressure was downsampled by a factor of 50 so that it could be imported and used. The decimation process is shown as follows:

(56) $$Y_{i}=\;X_{i*m+s}$$

For I = 0, 1, 2,…,size −1.

(57) $$size=\left(\frac{n-s}{m}\right)$$

where *n* is the number of elements in the input (undecimated data), *m* is the decimating factor (50), *s* is the start index (0), *and* size is the number of elements in the post-decimation results.

**Figure 8:**
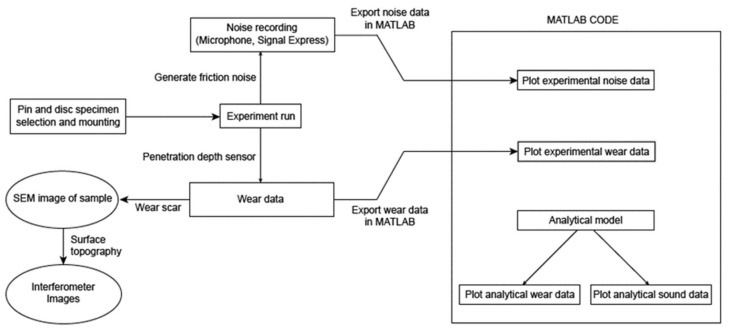
Flowchart of the experimental scheme.

**Figure 9:**
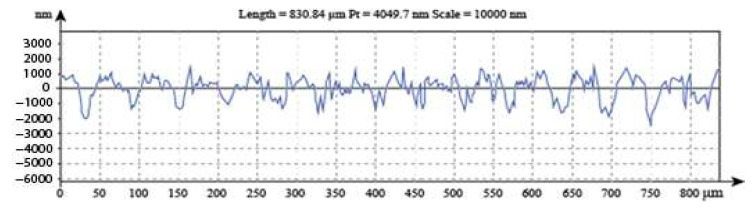
Surface roughness profile for the mild steel sample.

**Figure 10:**
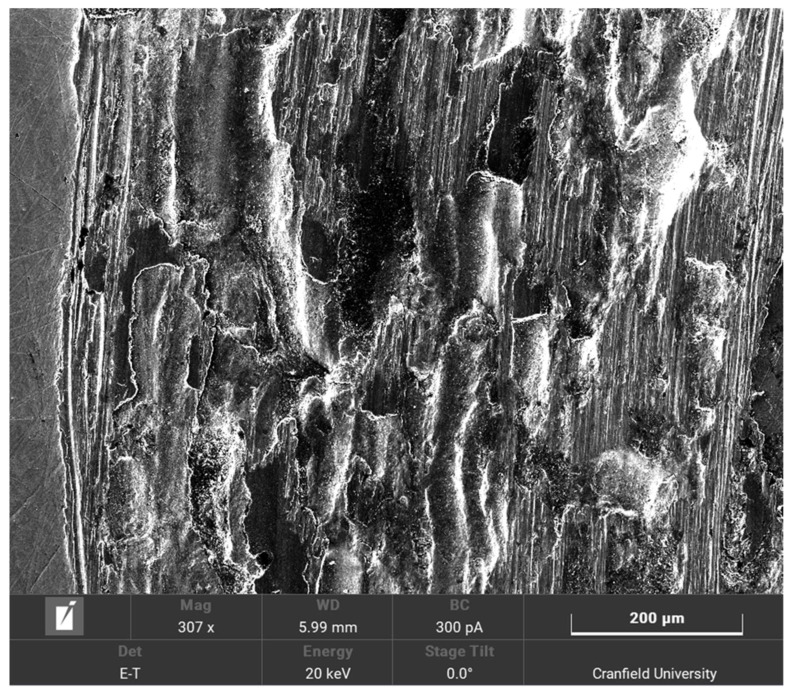
SEM image of the steel sample.

A set of SEM images are also taken after the experiments on a sample of discs. Further, interferometer images are also taken to visualise the surface roughness of the samples. The data for the sound pressure and the wear are imported into MATLAB for them to run alongside the theoretical model. Furthermore, an interferometer image is taken for some of the samples. A flowchart of the experimental scheme is shown in Figure 8:

One of the results from the interferometer for a mild disc sample is shown in Figure 9:

The surface roughness profile on the sample suggests that an exponential distribution is appropriate to model the asperity height distributions. As such, in the theoretical model, *Φ* (*y*, *t*) is assumed to be exponential and the mean value recorded by the interferometer was used in the original exponential distribution. A Gaussian distribution would be reflected by a smoother curve than the one shown in Figure 8 and the rougher the curve is, the closer it is to an exponential distribution [29]. The image from the scanning electron microscope is shown in Figure 10.

Figure 10 shows that the samples have undergone extensive wear. This suggests that the main mechanism for the sound generation is the wear caused by the breaking of the asperities. The plastic deformation does not contribute to sound generation.

## 4. Results and Discussion

### 4.1. Predicted and Observed Sound Pressure

**Figure 11:**
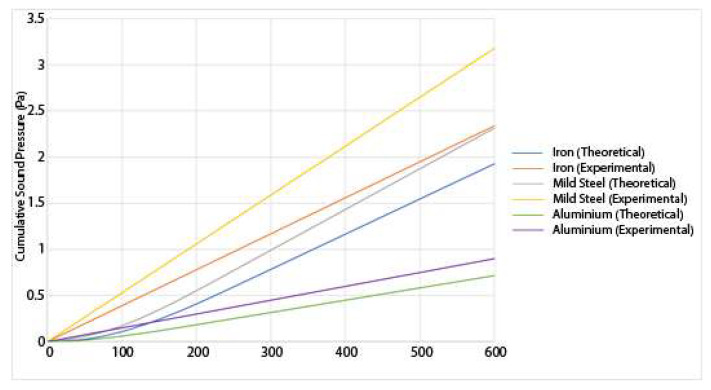
Experimental and theoretical results for the sound generation of various materials undergoing sliding friction under a 10 N load.

**Figure 12:**
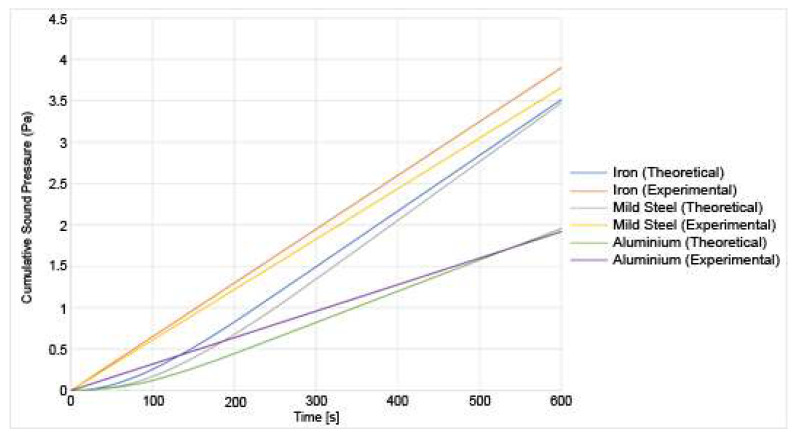
Experimental and theoretical results for the sound generation of varied materials undergoing sliding friction under a 20 N load.

The sound pressure is recorded for each of the three materials at the two different loads, and the results for both the theoretical and the experimental results are shown in Figure 11 and Figure 12:

From Figure 11 and Figure 12, a few numbers of different results can be deduced. First, the sound pressure generated is higher under a higher load than under a lower load. This is consistent with the existing research [7]. A higher sound generation is correlated to higher wear [3], therefore, it is reasonable to assume that as the sound increases, so does the wear. Both the experimental and theoretical results show a good correlation. Since the sound pressure is computed in the analytical model using the wear, the magnitude of the error is similar for the sound pressure as it is for the wear. This is because the sound pressure is dependent on the wear. The sound increase is mostly linear as the experiment goes on. It should be noted that the sound pressure shown in those results is not the sound pressure at any instant time, but the total cumulative sound pressure generated by the friction process. In those three materials, another conclusion that can be drawn is that iron generates the higher sound pressure at 20 N followed, by mild steel. Aluminium generates the lowest amount of noise. This is because the aluminium is wear-resistant due to its heat treatment. The asperities spend a longer period in the elastic zone due to the high tensile strength. Due to a lower number of breaking asperities, the sound produced by the aluminium disc is lower than for the other materials. This also correlates with lower wear. The results also show that the amount of sound produced by the theoretical model has been underestimated. This could be because there are other sources of sound produced in the experiment that the model does not account for such as any external vibrations of the setup. The wear has also been underestimated in the theoretical model. Since the wear is a major contributor in the production of sound, then that means that if the wear has been underestimated, then the sound produced is too.

### 4.2. Predicted and Observed Wear

**Figure 13:**
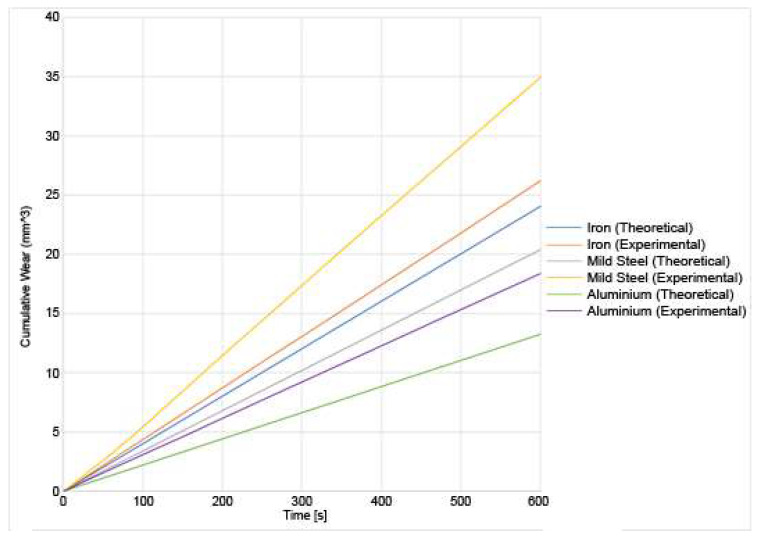
Experimental and theoretical results for the wear of various materials under a 10 N load.

**Figure 14:**
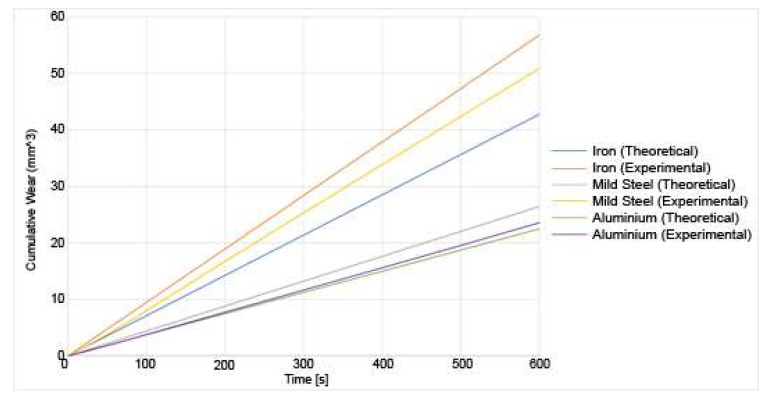
Experimental and theoretical results for the wear of varied materials under a 20 N load.

The experimental and theoretical results for the wear of the various materials and under the different loads are shown in Figure 13 and Figure 14:

From those results, out of the three materials, iron undergoes the most wear at a higher load, both from a theoretical and experimental viewpoint. Aluminium T351 undergoes the least wear. This is expected as heat-treated aluminium is more wear-resistant than either pure iron or mild steel. It can also be observed that the wear increases with increasing load [30]. The errors are calculated between the observed wear and the predicted wear. They are around 10% for aluminium, 15% for iron and 30% for mild steel, which shows the larger error. The wear has been underestimated in the theoretical model. This could be due to several factors. First, the model was simplified. For example, the major assumption made is that the impact is a point impact on the asperities. However, this would not be the case in a real setup. The impacts would be a distributed impact across the asperity. However, modelling that would require a finite-element analysis on each asperity to determine how the force would be distributed and is not done as it would be too computationally expensive. Similarly, the top asperity is assumed to be a single asperity contacting with one asperity at a time on the bottom surface. However, in the experimental setup, the pin would be made from hundreds of asperities all interacting with hundreds of asperities on the bottom. This could be incorporated into this model as a refinement of the existing model. Moreover, the asperities were assumed to be independent. This resulted in an underestimate for the wear, as in a real asperitical distribution, it is possible that one asperity could impact the neighbouring asperity, thus causing a higher force than was estimated in the model. Finally, the asperities are modelled as cantilever beams out of simplicity. Therefore, they are assumed to behave as macroscopic cantilever beams. There are grounds for refinements that would improve the accuracy. Assuming an asperitical distribution for both the top asperities and the bottom asperities would account for more interactions that happen during the friction processes. Furthermore, other assumptions could be refined. For example, the current model assumes that the top asperity causes a point impact on the bottom asperity and that the whole asperity breaks off regardless of the point of impact. After which, a new asperity is generated in its place. A potential improvement would be to calculate the point at which the asperity would break. The height of the new asperity would therefore be the remaining height of the previous asperity. The frequency field of the sound spectrum was also not analysed in this research. Only the sound pressure was recorded. The frequency field would allow the determination of what frequencies contribute to the acoustic radiation along with how the dominant frequencies would change as wear progresses. Moreover, the scale of the model could be refined. Material properties are scale-dependant. Bulk material properties were used in the model as the asperities were on a mesoscale, so it was assumed that the bulk material properties would hold sufficiently well. However, improvements could be made to incorporate the effect of wear debris between the cracks in the asperities. Though complicated to model, ambient humidity could also have an effect. At sufficiently small scales, water droplets formed from ambient condensation would form a meniscus in the cracks of the contact zones between the surfaces. Finally, the effect of flash temperatures was not implemented in the model.

## 5. Conclusions

This paper presents a theoretical approach to predict the wear volume and the sound pressure generated during a sliding process for various materials under different loads. The current approach implies that only two mechanisms are responsible for the generation of sound. The first mechanism is the elastic vibration of the asperities, and the second mechanism is the energy released during the breaking of the asperities. The results show that as the total sound pressure increases, so does the wear volume. This leads to the possibility of quantifying the wear present in the material by analysing the cumulated sound pressure that occurs during the sliding process. The theoretical model only requires the material properties to compute the wear and the predicted sound pressure which makes it a simple model to use in practical applications. However, there are several drawbacks to the model that limits its accuracy. For example, the initial assumption that the top surface is a single asperity causing a point impact on each disc asperity is an oversimplification that leads to the wear being underestimated. Similarly, the sound pressure estimated by the model has also been underestimated, although the error margin is less. Overall, the theoretical results qualitatively agree with the experimental results. This means that after further refining the model, such as by considering a distributed force of impact or lubrication, the ability to predict the wear generation based on the sound pressure generated could provide a non-destructive means of assessing the wear of materials.

## Data Availability

The data presented in this study are available on request from the corresponding author.

## References

[1] Holmberg K., Erdemir A. (2017). Influence of tribology on global energy consumption, costs and emissions. Friction.

[2] Yigezu B.S., Jha P.K., Mahapatra M.M. (2013). Effect of Sliding Distance, Applied Load, and Weight Percentage of Reinforcement on the Abrasive Wear Properties of In Situ Synthesized Al-12%Si/TiC Composites. Tribol. Trans..

[3] Khan M., Basit K., Khan S., Khan K., Starr A. (2017). Experimental assessment of multiple contact wear using airborne noise under dry and lubricated conditions. Proc. Inst. Mech. Eng. Part J J. Eng. Tribol..

[4] Finkin E.F. (1978). An explanation of the wear of metals. Wear.

[5] Yokoi M., Nakai M. (1982). A Fundamental Study on Frictional Noise: (5th Report, The influence of random surface roughness on frictional noise). Bull. JSME.

[6] Jacobson S., Heldi M., Heinrichs J. (2021). On the critical roles of initial plastic deformation and material transfer on the sliding friction between metals. Wear.

[7] Abdelounis H.B., le Bot A., Zahouani H., Perret-Llaudet J. Experimental Study on Friction Noise of Dry Rough Surfaces. Proceedings of the STLE/ASME 2008 International Joint Tribology Conference.

[8] Bakar A.R.A., Ouyang H., James S., Li L. (2008). Finite element analysis of wear and its effect on squeal generation. Proc. Inst. Mech. Eng. Part D J. Automob. Eng..

[9] Boness R.J., McBride S.L., Sobczyk M. (1990). Wear Studies using acoustic emission techniques. Tribol. Int..

[10] Boness R.J., McBride S.L. (1991). Adhesive and abrasive wear studies using acoustic emission techniques. Wear.

[11] Benabdallah H.S., Aguilar D.A. (2008). Acoustic Emission and Its Relationship with Friction and Wear for Sliding Contact. Tribol. Trans..

[12] de Moerlooze K., Al-Bender F., van Brussel H. (2010). A Generalised Asperity-Based Friction Model. Tribol. Lett..

[13] Eriten M., Polycarpou A.A., Bergman L.A. A physics-based fretting model with friction and integration to a simple dynamical system. Proceedings of the ASME 2011 International Design Engineering Technical Conferences & Computers and Information in Engineering Conference.

[14] Emami A., Khaleghian S., Su C., Taheri S. Physics-Based friction model with potential application in numerical models for tire-road traction. Proceedings of the Dynamic Systems and Control Conference.

[15] Liu T., Liu G., Xie Q., Wang Q.J. (2006). Two-Dimensional Adaptive-Surface Elasto-plastic Asperity Contact Model. J. Tribol..

[16] Savio G., Meneghello R., Concheri G. (2009). A surface roughness predictive model in deterministic polishing of ground glass moulds. Int. J. Mach. Tools Manuf..

[17] Quinn T.F. (1971). Oxidational wear. Wear.

[18] Shen X., Cao L., Ruyan L. Numerical simulation of sliding wear based on archard model. Proceedings of the 2010 International Conference on Mechanic Automation and Control Engineering.

[19] Hassan A.K.F., Mohammed S. (2016). Artificial Neural Network Model for estimation of wear and temperature in pin-disc contact. Univ. J. Mech. Eng..

[20] Fillot N., Iordanoff I., Berthier Y. (2007). Wear modeling and the third body concept. Wear.

[21] Bengisu M.T., Akay A. (1999). Stick-Slip oscillations: Dynamics of friction and surface roughness. J. Acoust. Soc. Am..

[22] Blau P.J. (2013). Asperities. Encyclopedia of Tribology.

[23] Greenwood J.A., Williamson J.B. (1966). Contact of nominally flat surfaces. Proc. R. Soc. A Math. Phys. Sci..

[24] Stoyanov P., Chromik R.R. (2017). Scaling Effect on Materials Tribology: From Macro to Micro Scale Materials. Materials.

[25] Volterra E., Zachmanoglou E.C., Kolsky H. (1966). Dynamics of Vibrations. J. Appl. Mech..

[26] Gere J.M., Goodno B.J. (2012). Mechanics of Materials.

[27] Norton M.P., Pan J. (2001). Noise Radiated by Baffled Plates. Encyclopedia of Vibration.

[28] Gohar R., Rahnejat H. (2012). Fundamentals of Tribology.

[29] Dierking W. (1999). Quantitative Roughness Characterization of Geological Surfaces and Implications for Radar Signature Analysis. IEEE Trans. Geosci. Remote Sens..

[30] Mazahery A., Shabani M.O. (2012). Study on microstructure and abrasive wear behaviour of sintered Al matrix composites. Ceram. Int..

